# B-cell translocation gene 2 mediates crosstalk between PI3K/Akt1 and NFκB pathways which enhances transcription of MnSOD by accelerating IκBα degradation in normal and cancer cells

**DOI:** 10.1186/1478-811X-11-69

**Published:** 2013-09-18

**Authors:** Santhoshkumar Sundaramoorthy, Min Sook Ryu, In Kyoung Lim

**Affiliations:** 1 Department of Biochemistry and Molecular Biology, BK21 Cell Transformation and Restoration, Ajou University School of Medicine, Suwon 443-721, Republic of Korea

**Keywords:** BTG2/TIS21/Pc3 gene, MnSOD, Akt1, NFκB, IκBα, G2/M arrest

## Abstract

**Background:**

B-cell translocation gene 2 (BTG2) belongs to antiproliferative (ARPO) gene family and the expression of BTG2, human ortholog of rat PC3 and mouse TIS21 gene, has been shown to render cancer cells more sensitive to doxorubicin treatment by upregulating MnSOD expression without regulating any other reactive oxygen species (ROS) scavenging enzymes.

**Results:**

In the present study, by employing exogenous and endogenous BTG2^/TIS21/Pc3^ expression by transfection and transduction analyses, and by knockdown of gene expression using RNA interference or using gene knockout cells, we observed that BTG2 increased the binding of activated NF-κB (p65/RelA) to the enhancer element of MnSOD gene in the 2^nd^ intron, which was regulated by p-Akt1, and the induction of MnSOD by BTG2 was accompanied with subsequent downregulation of ROS level and cyclin B1 biosynthesis along with the increase of p21^WAF1^, resulting in the G2/M arrest independent of p53.

**Conclusions:**

These results show for the first time that BTG2 mediates crosstalk between PI3K-Akt1 and NF-κB pathways, which regulates p53-independent induction of G2/M phase arrest both in normal and cancer cells.

## Lay abstract

### Background

B-cell translocation gene 2 (BTG2) belongs to the anti-proliferative gene family, whose expression is deregulated in carcinogenesis of numerous tissues such as prostate, kidney, breast, liver and brain.

### Results

Degradation of IκBα was accelerated via activation of Akt1 in the cells with BTG2 over-expressed, suggesting the regulation of crosstalk between PI3K/Akt1 and NFκB pathways.

### Conclusion

Activated NFκB binds to the enhancer of MnSOD gene and activates its transcription in the cells with BTG2 expression.

### Significance

Learning how BTG2 up-regulates MnSOD is important for understanding the antioxidant and the tumor suppressive roles of B Cell Translocation Gene 2 in normal and cancer cells.

## Background

Human BTG2 belongs to the antiproliferative (APRO) gene family along with its orthologs, rat PC3 and mouse TIS21 [1]. It potentiates NGF-induced differentiation of PC12 cells and protects neurons from apoptosis [2]. In contrast, overexpression of BTG2 significantly enhances reactive oxygen species (ROS) generation after doxorubicin treatment of HeLa cells via burst of H_2_O_2_ by induced manganese-containing superoxide dismutase (MnSOD) without regulating catalase and glutathione peroxidase expression [3]. The APRO gene, including TIS21 [4], PC3 [5] and BTG2 [6], has originally been reported as a primary response gene transiently expressed in the established cell lines in response to various stimulations, however, BTG2^/TIS21/PC3^ (BTG2) expression in mouse and human tissues has been shown to be rather constitutive [7], and it is found to be significantly reduced during carcinogenesis in the epithelial cells of thymus [8], prostate [9], kidney [10], breast [11], liver [12] and brain [13]. Furthermore, the loss of BTG2 expression is related significantly with tumor grade, metastasis and resistance to cancer treatment, especially in the estrogen receptor positive breast cancers [14]. In addition, BTG2 regulates cancer cell migration via regulations of ROS level and chemokines Cxcl3 and Cxcl12 [13,15]. It has been reported that BTG2 plays a major role at the downstream of p53 gene [16], inhibits Ras-induced cell transformation [17] by inhibiting its activation to RasGTP via direct interaction with Ras protein [18]. Therefore, BTG2 is also involved in the regulation of mutant p53 activities via inhibiting Ras-related cancer gene signatures [19]. All the above mentioned findings together strongly suggest the important roles of BTG2 played in carcinogenesis. BTG2 also regulates both G1/S and G2/M phase arrest independent of p53 and pRB expression; G1/S phase arrest is regulated by inhibiting synthesis of cyclin E and cdk4 activity independent of pRB [20], whereas BTG2 regulates G1/S progression in pRB dependent manner by reducing cyclin D1 expression [21]. Furthermore, BTG2 strongly induces G2/M phase arrest in U937 myelomonocytic leukemia (p53 null) and Huh7 (p53 mutant) hepatoma cells [22], therefore, we earlier suggested BTG2 as a pan-cell cycle inhibitor independent of p53 and pRB activities via interaction with Pin1 in response to EGF stimulation [23].

Several reports suggest that BTG2 protein is a general activator of mRNA deadenylation. BTG2 overexpression caused accelerated deadenylation of reporters and of endogenous transcripts. It directly interacts with Caf1 which is a component of the CCR4–NOT deadenylase complex. These observations for the first time showed the role of BTG2 in the general control of mRNA decay [24-26]. As mentioned above, BTG2 enhances MnSOD expression in HeLa cells after treatment with doxorubicin [3] and MnSOD has been suggested as a new type of tumor suppressor [27]. Overexpression of MnSOD inhibits the growth of a wide variety of cancer cells and protects oxygen-utilizing cells from the toxicity of ROS under physiologic condition [28]. Furthermore, MnSOD expression is regulated at the transcriptional, translational and posttranslational levels, and Sp1 and AP-2 are reported to be homeotic transcriptional regulators of MnSOD [29], whereas induction of MnSOD expression requires the binding of NF-κB transcription factor (p65) to the enhancer element in the intron-2 of MnSOD gene [30]. In addition, acetylation of MnSOD influences its enzymatic activity in response to nutrient status or oxidative stress at the posttranslational level [31].

In the present study, we attempted to answer the questions of whether MnSOD can be a direct transcriptional target of BTG2 or not, what is the mechanism of MnSOD induction by BTG2, what kinds of signal pathways regulate the induction of MnSOD expression, and what is outcome of MnSOD induction by BTG2 in cancer and normal cells. In fact, various lines of evidences suggest that free radical-scavenging agents inhibit neoplastic processes at the cellular and molecular levels [32]. Tumor cells are low in MnSOD activity and its expression, compared to normal counterparts [27], therefore, tumor cells may particularly be able to accumulate higher levels of superoxide or hydrogen peroxide than the normal, which may enhance survival and proliferation of the cancer cells. We observed in the present study that the expression of MnSOD was upregulated by BTG2 at the transcription level via degradation of IκBα in cancer and normal cells, and that underlying mechanism of APRO effect of BTG2 involved the crosstalk between PI3K-Akt1 and NFκB pathways, which resulted in the scavenge of ROS and increased p21^WAF1^ expression.

## Results

### BTG2 upregulates MnSOD expression in HeLa cells

**Figure 1:**
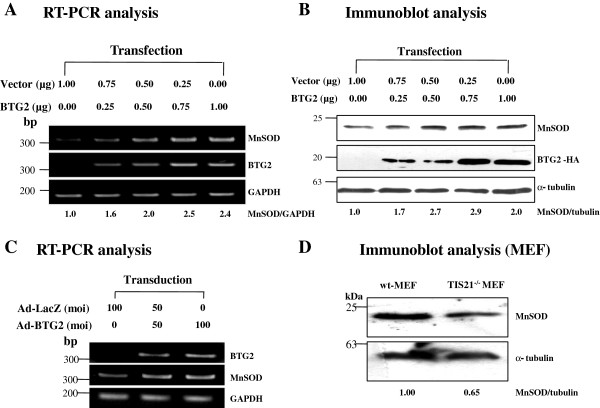
**Upregulation of MnSOD expression in HeLa cells by BTG2**^**/TIS21**^**. (A)** Transfection analysis; HeLa cells (2×10^5^ cells in 60 mm) were transfected with pcDNA3-TIS21(HA) for 6 h along with the empty vector (pcDNA3-HA) to adjust total DNA contents, and then subjected to RT-PCR analyses using the primers described in the Additional file 5. Expression of MnSOD was increased in the BTG2 overexpressed samples in a concentration dependent manner. GAPDH was employed as a control expression of transcription. **(B)** Expression of MnSOD was increased along with the overexpression of BTG2^/TIS21^, examined by immunoblot analysis. Tubulin expression was used for loading control in each lane. **(C)** Transduction analysis; Ad-BTG2 was infected to HeLa cells (2 × 10^5^ in 60 mm dish) for 5 h and then BTG2-mediated MnSOD expression was evaluated by RT-PCR. Expression of MnSOD was increased after Ad-BTG2 transduction. **(D)** To evaluate effect of endogenous BTG2^/TIS21^ gene on the expression of MnSOD, wild type-MEF and BTG2^/TIS21^ knockout-MEF lysates were subjected to immunoblot analysis. Endogenous level of MnSOD expression was lower in the TIS21^−/−^MEF than the wt-MEF, when measured by Image J software.

We earlier showed that BTG2 sensitizes HeLa cells to doxorubicin treatment via induction of endogenous MnSOD expression [3]. To investigate molecular mechanism involved in MnSOD induction by BTG2, we overexpressed BTG2 gene in HeLa cells by DNA transfection and viral transduction, and found that BTG2 upregulated MnSOD expression in the dose-dependent manner both in its mRNA (Figure 1A and 1C) and protein (Figure 1B) levels. On the other hand, endogenous level of MnSOD expression in the TIS21^−/−^ MEF along with wt-MEF was much lower in the TIS21^−/−^ MEF than the control (Figure 1D), suggesting that the effect of BTG2 on MnSOD expression was both endogenously and exogenously regulated in mouse fibroblasts and cancer cells, and that there is a possibility of MnSOD gene as a direct target of BTG2.

### BTG2 activates NFκB-response element of MnSOD gene in the 2nd intron

**Figure 2:**
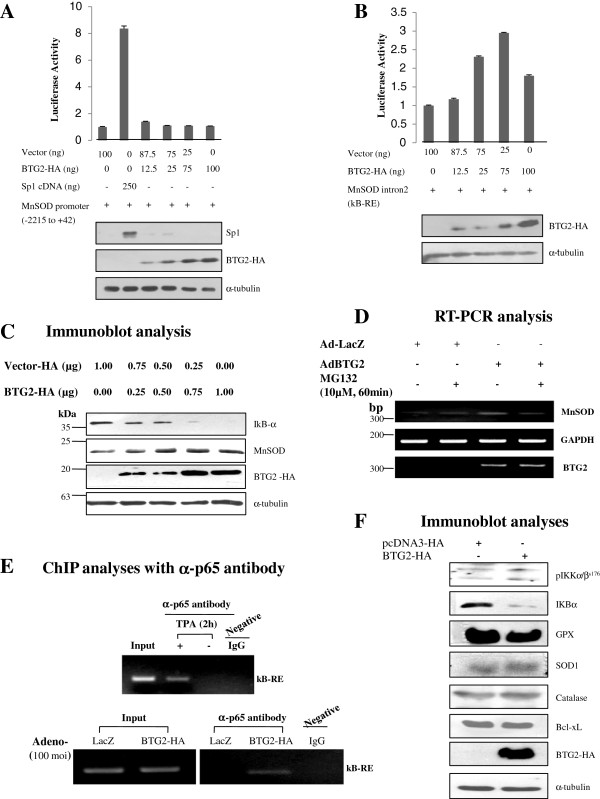
**Activation of NFκB-response element in the 2**^**nd **^**intron of MnSOD gene by BTG2. (A)** MnSOD promoter analysis; Cells (0.5×10^5^/12 wells) were cotransfected with BTG2 cDNA and promoter DNA of MnSOD gene and then subjected to luciferase analysis. Transfection of BTG2 up to 100 ng failed to activate promoter of MnSOD. Sp1 cDNA was employed as a positive control. Lower panel shows protein expressions of the transfected DNAs and loading control. **(B)** Cells were cotransfected with BTG2 cDNA and the κB-RE before luciferase assay. Expression of MnSOD was significantly increased with transfection of BTG2, indicating the activation of κB-RE by BTG2. Immunoblot analysis shows protein expression of BTG2-HA and loading control. **(C)** To further investigate the regulation of NFκB activation and MnSOD induction by BTG2 expression, IκBα degradation was examined by immunoblot analysis. Note the degradation of IκBα and MnSOD expression in the BTG2-dependent manner. **(D)** Transduction of HeLa cells with Ad-BTG2 was performed and then treated with 10 μM of MG132 for 1 h before RT-PCR analysis. MG132 abolished induction of MnSOD expression by BTG2, suggesting the upregulation of MnSOD expression by BTG2 via proteasomal degradation of IκBα. **(E)** Cell lysates with the LacZ or the BTG2 expressers were subjected to ChIP analysis with anti-p65 antibody, and then the interaction was verified by PCR reaction with the primers written in the Additional file 5. To verify our analysis, HeLa cells were treated with or without 100 ng of 12-*O*-tetradecanoylphorbol-13-acetate (TPA) for 2 h and then applied to ChIP assay as a positive and negative control. Unstimulated IgG was employed to exclude the nonspecific interaction. Inputs indicate total amount of κB-RE present in the samples. Note the interactions of p65 with κB-RE only in the BTG2 expressers and the TPA treated positive cells. **(F)** Immunoblot analyses showing the activation of IKKα/β in the BTG2 overexpressers without any changes in the expression of other anti-oxidant enzymes.

To investigate a mechanism of MnSOD induction by BTG2 in HeLa cells, we performed luciferase assay using the promoter DNA construct (−2215 to +42) of MnSOD gene ligated to the upstream of luciferase, however, there was no difference in the promoter activity by the expression of BTG2 gene (Figure 2A), except by Sp1, the positive control. Since inducible expression of MnSOD has been shown to be regulated by NFκB binding to the enhancer element of MnSOD gene [30], we subcloned the NF-κB response element (κB-RE) into the pGL3-BASIC vector and performed luciferase analysis with or without BTG2 coexpression. As shown in Figure 2B, the luciferase activity was increased with BTG2 coexpression, suggesting that BTG2 enhances the activity of κB-RE in the MnSOD gene. Proteins of the transfected genes were monitored by immunoblot analyses and presented under the Figure 2A and 2B. To rule out other possibility of MnSOD induction by BTG2 expression, the activity of FOXO3a, a well known transcription factor of MnSOD in quiescent cells against oxidative stress [33], was analyzed by measuring its phosphorylation on serine253 residue, however, there was no significant difference of its activity after BTG2 expression (Additional file 1: Figure S1A). To further investigate the BTG2-mediated activation of κB-RE of MnSOD gene, the activation of NFκB pathway in HeLa cells was evaluated by measuring the degradation of IκBα in proteasome [34]. Increasing amounts of BTG2 expression significantly enhanced the degradation of IκBα protein along with MnSOD expression (Figure 2C). Moreover, when the cells were treated with MG132 to block ubiquitin proteasome pathway, BTG2-mediated MnSOD induction was accordingly reduced (Figure 2D). The data support our observation in Figure 2B that BTG2 increased transcription of MnSOD through the activation of κB-RE. To further confirm the activation of NFκB by BTG2, we performed ChIP analysis using the cell lysates obtained from the BTG2 and the LacZ expressers. As expected, the interaction of p65 with κB-RE was observed only in the BTG2 expresser, but not in the control (Figure 2E, lower panel). Since TPA has been known to induce MnSOD through NFκB pathway [35,36], TPA treatment (100 ng/mL for 2 h) was employed as a positive control for the ChIP analysis. As expected, TPA increased IKBα degradation (Additional file 1: Figure S1B) and induced binding of p65 to κB-RE of MnSOD gene (Figure 2E, upper panel). To further evaluate the upstream kinase of IκBα, phosphorylation of IKKα/β on serine176 residue was examined, and its minimal activation was observed along with significant degradation of IκBα in the BTG2 expresser without any other antioxidant scavenging enzymes such as catalase, GPX, or SOD1 (Figure 2F).

### BTG2 mediated–NFκB activation is regulated by IκBα degradation

**Figure 3:**
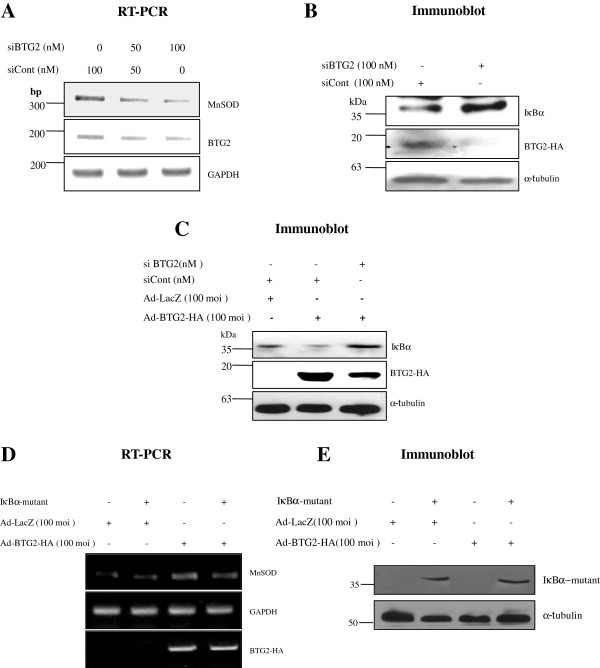
**Regulation of BTG2 mediated–NFκB activation by IκBα degradation. (A)** To confirm the effect of BTG2 on the activation of NFκB and its regulation of MnSOD induction, the mixture of five short interfering RNAs to BTG2 (siBTG2#1 - siBTG2#5) was transfected to HeLa cells for 48 h and then expression of MnSOD was analyzed by RT-PCR. The expression was downregulated by the concentration of siBTG2 dependently, indicating the regulation of MnSOD expression by endogenous BTG2. The sense and the antisense primer sequences of siBTG2 were described in the Additional file 6. **(B)** Immunoblot analysis revealing the regulation of IκBα degradation by BTG2. Knockdown of endogenous BTG2 expression by transfection of HeLa cells with 100 nM siBTG2 abolished BTG2 protein expression, in contrast to the accumulation of IκBα. **(C)** Transfection of siBTG2 blocked the effect of exogenous BTG2^/TIS21^ on IκBα degradation, indicating the same effect of the exogenous BTG2^/TIS21^ and the endogenous BTG2 genes on the regulation of NFκB activation. **(D)** To further evaluate the effect of BTG2^/TIS21^ gene on the IκBα degradation and the upregulation of MnSOD expression, HeLa cells were infected with lentivirus containing IκBα mutant (Ser^32, 36^Ala) prepared in our laboratory. Overexpression of the mutant reduced upregulation of MnSOD transcription stimulated by BTG2 gene. **(E)** Complete failure of the BTG2-mediated degradation of IκBα mutant (Ser^32, 36^Ala), examined by immunoblot analysis.

To further confirm the specificity of BTG2 effect on MnSOD expression via NFκB activation, HeLa cells were transfected with short interfering RNAs against BTG2 (siBTG2), and the changes of MnSOD and IκBα expression were evaluated by RT-PCR and immunoblot analyses, respectively. Knockdown of endogenous BTG2 downregulated MnSOD level (Figure 3A), whereas IκBα was further accumulated (Figure 3B). Moreover, transfection of siBTG2 also reduced the effect of exogenous BTG2 on the degradation of IκBα protein in the cells (Figure 3C). To confirm the effect of IκBα degradation on the BTG2-regulated MnSOD induction, successive transductions of the lentivirus with nondegradable IκBα mutant and the Ad-BTG2-HA were performed. As shown in Figure 3D, the BTG2-induced MnSOD expression was reduced by coinfection with IκBα-mutant which was unable to be disrupted (Figure 3E), supporting the activation of NFκB by BTG2 is specific and mediated via IκBα degradation.

### BTG2-enhanced IκBα degradation is regulated by p-Akt1

**Figure 4:**
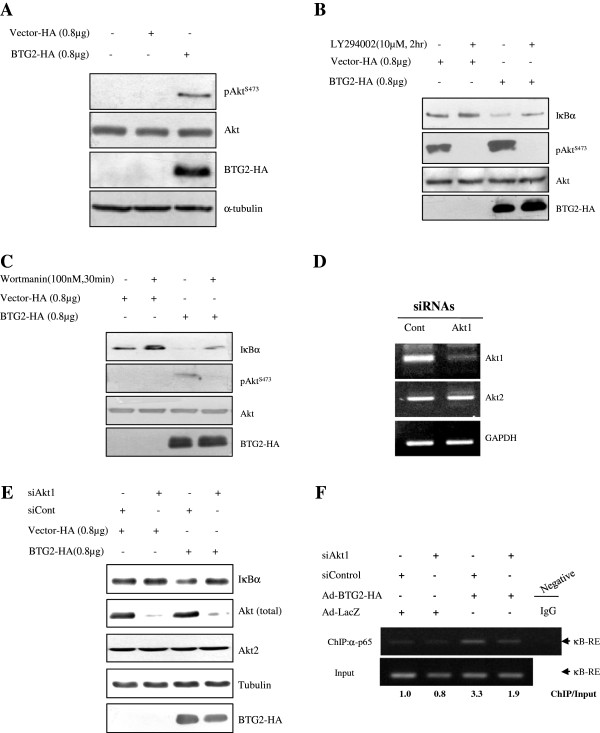
**Regulation of BTG2-enhanced IκBα degradation via activation of PI3K-Akt1.** Based on our previous report [37], upstream signals of the IκBα degradation by BTG2 were investigated. **(A)** Immunoblot analysis showing the significant phosphorylation of Akt at Ser^473^ residue in the BTG2 expresser. To explore whether there is a crosstalk between PI3K and NFkB pathways, HeLa cells transfected with BTG2 were treated with PI3K inhibitors, LY294002 **(B)** and Wortmanin **(C)**, respectively, and then regulation of IκBα degradation was examined by immunoblot analyses. Note significant inhibition of BTG2-mediated IκBα degradation after treatment with PI3K inhibitors, suggesting the cross-talk between PI3K-Akt and NFκB pathways by the expression of BTG2. **(D)** RT-PCR revealing the specific knockdown of Akt1, not Akt2, by the short interfering RNAs to Akt1. **(E)** BTG2-mediated IκBα degradation was significantly inhibited by transfection of siAkt1, whereas transfection of siControl failed to change IκBα degradation at all, supporting the specific effect of Akt1 on the activation of BTG2-mediated NFκB pathway. **(F)** To further specify the effect of Akt1 on the BTG2-mediated IκBα degradation, transfection of siAkt1 RNA was combined with Ad-BTG2 infection in HeLa cells, and then ChIP analysis was performed with anti-p65 antibody. As expected, interaction of p65 with κB-RE was significantly reduced in the BTG2 and siAkt1 coexpressers (1.9 *vs.* LacZ and siControl) than that of the BTG2 alone expresser (3.3 *vs.* LacZ and siControl), indicating downregulation of NFκB activation by siAkt1 (over 40%) and the activity of Akt1 at the upstream of NFkB activation in the presence of BTG2 expression. Inhibition of p65 binding to κB-RE after transfection of siAkt1 was quantified by Image J software, and the relative densities of kB-RE found in the ChIP assay based on those of the Input (ChIP/Input) were showed below the Figure 4F. The experiment was repeated (n = 3).

We have earlier shown that the activation of Akt in response to estradiol requires BTG2 expression in MEF and bone marrow precursor cells (Lin^-^-c-Kit^+^-Sca1^+^) [37]. Therefore, the possibility of crosstalk between PI3K-Akt and NFκB pathways was explored. When HeLa cells were transfected with BTG2, Akt phosphorylation on Serine473 was significantly increased (Figure 4A). To further explore the crosstalk between pAkt and IκBα degradation, PI3K inhibitors (LY294002 and Wortmanin) were applied to the test system and the effect of PI3K inactivation on the expression of IκBα and Akt activation was examined. The result in the 3^rd^ and 4^th^ lanes in Figure 4B and 4C clearly showed the regulation of NFκB activation by PI3K-Akt signal. To further specify the role of Akt1 in the activation of NFκB pathway, HeLa cells were transfected with siAkt1 (Figure 4D) and the inhibition of BTG2-mediated IκBα degradation was examined by immunoblot analysis. As expected, knockdown of Akt1 recovered BTG2-mediated degradation of IκBα expression in the siAkt1 and BTG2 cotransfected cells (Figure 4E). Moreover, ChIP assay revealed inhibition of p65 binding to κB-RE up to 40% by cotransfection of siAkt1 and BTG2 (3^rd^ and 4^th^ lanes in Figure 4F). These data strongly suggest the BTG2-mediated Akt1 activation at the upstream of IκBα degradation.

**Figure 5:**
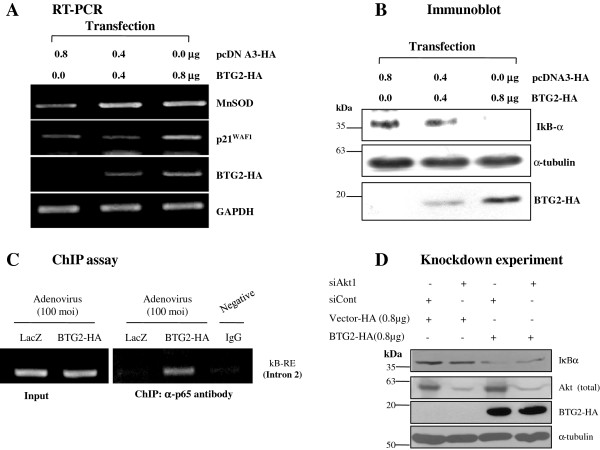
**BTG2-mediated MnSOD expression via crosstalk of Akt1 and NFκB in A549 cells.** To further investigate the regulation of MnSOD expression by BTG2 via activations of Akt1 and NFκB pathways in addition to HeLa cells, A549 human lung cancer cells were subjected to transfection of BTG2^/TIS21^ and adenoviral transduction analyses for the following experiments. **(A)** RT-PCR analysis showing the increased expression of MnSOD gene in A549 cells after overexpression of BTG2. **(B)** Immunoblot analysis showing the degradation of IκBα after transcfection of BTG2 for 48 h. **(C)** ChIP assay revealing the interaction of p65 with κB-RE in the MnSOD gene in the BTG2 overexpressed cells. **(D)** Activation of NFκB pathway by Akt1 in the BTG2 transfected A549 cells. Degradation of IκBα in the BTG2 expresser was reduced by transfection of siAtk1 to A549 cells for 48 h.

To exclude the possibility that the crosstalk is a phenomenon limited to HeLa cells, A549 human lung cancer cells were employed and confirmed the induction of MnSOD by transfection of BTG2 (Figure 5A) with concomitant degradation of IκBα (Figure 5B). Moreover, the specific binding of p65 to κB-RE was also observed in the BTG2 expressers by ChIP assay (Figure 5C). At the same time, the crosstalk between pAtk1 and NFκB was confirmed by employing knockdown of Akt1 in the same cells (Figure 5D). Furthermore, BTG2 mediated-IκBα degradation was confirmed also in MCF7 breast cancer cells and wt-MEF after transfections of BTG2 cDNAs and siBTG2^/TIS21^, respectively (Additional file 2: Figure S2). All these data strongly suggest the regulation loop between BTG2-PI3K/Akt1-NFκB pathways for the induction of MnSOD expression.

### BTG2 enhances G2/M arrest along with reduction of H_2_O_2_ level

**Figure 6:**
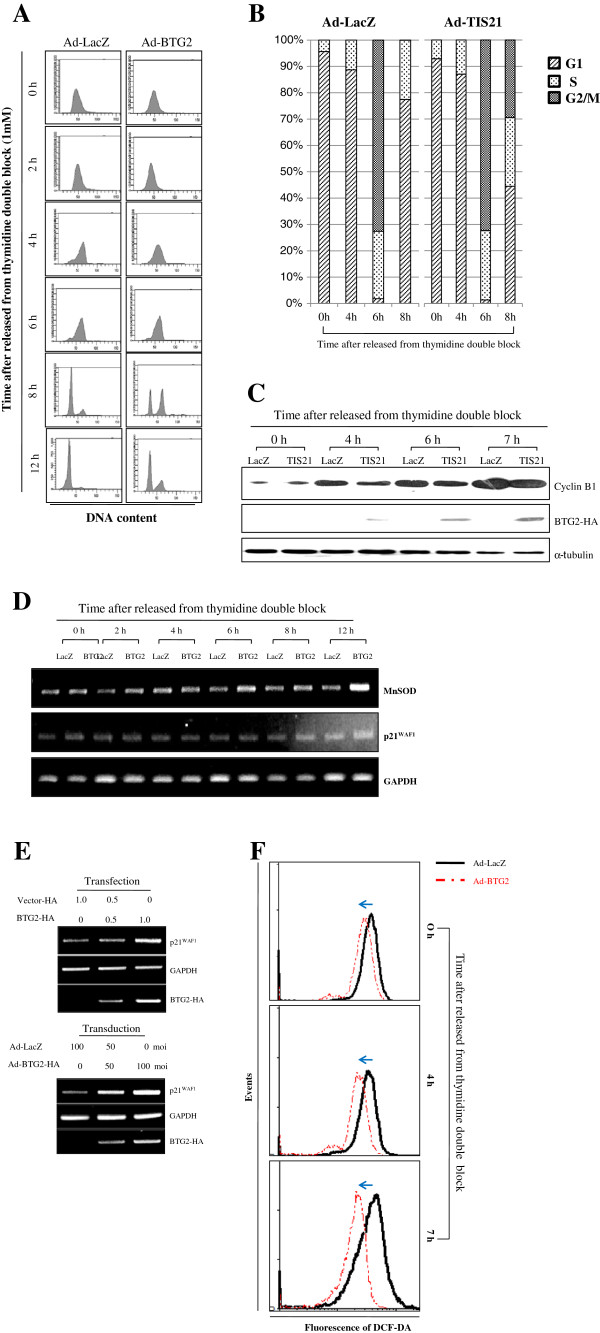
**G2/M arrest along with reduction of H**_**2**_**O**_**2 **_**level in the BTG2 expresser.** HeLa cells were synchronized at G1/ S boundary by thymidine double block (TDB) before the following experiments. **(A)** Cell cycle analysis; Cells were harvested at the indicated times after TDB for FACS analyses. The X-axis corresponds to DNA content and Y-axis indicates number of cells. Note significant G2/M arrest in the BTG2 expresser at 8 h compared with the LacZ. **(B)** The cells in each phase were analyzed by ModFit software, and the statistical difference between the LacZ and the BTG2 expressers were evaluated by paired *t*-test. Note statistical significance in the G1 phase (p = 0.04) and the G2/M phase (p = 0.03) cells in the BTG2 expresser compared to LacZ control at 8 h. **(C)** Immunoblot analysis; Delayed synthesis of cyclin B1 in the BTG2 overexpresser at 4 h after TDB. **(D)** RT-PCR analysis showing the increased expressions of MnSOD and p21^WAF1^ in the BTG2 overexpresser at 6 h and at 8 h after TDB, respectively. **(E)** Induction of p21^WAF1^ expression in HeLa cells overexpressing BTG2 gene, proved by transfection and transduction of BTG2 gene. **(F)** Reduction of intracellular H_2_O_2_ in the BTG2 overexpresser than the LacZ control. HeLa cells were treated with 20 μM DCFDA for 15 min and analyzed by FACS. Note significant reduction of H_2_O_2_ level in the BTG2^/TIS21^ expresser than the LacZ control at 7 h after TDB.

To explore physiologic significance of BTG2 overexpression, HeLa cells were synchronized at the late G1 and early S phases by thymidine double blocks and the cell cycle progression was monitored for 12 h by FACS analysis after the release. Figures 6A (X-axis corresponds to DNA content and Y-axis represents number of event) and 6C revealed that the expression of cyclin B1 was delayed along with G2/M arrest in the BTG2 expressers at 4 h after the release and the phenomenon is well accordant with our previous report observed in U937 cells [22]. When the percentage of cells in G1 and G2 phases were calculated using the ModFit software, Figure 6B clearly revealed G2/M arrest in the BTG2 expresser, compared with the LacZ control.To further evaluate whether the G2/M arrest in the BTG2 expresser is restricted only to cancer cells or not, we analyzed cell cycle progression of NIH3T3 cells infected with adenovirus carrying either BTG2 or LacZ control and synchronized at G1/S phases by using thymidine double block (Additional file 3: Figure S3A). There was no significant differences of G2/M phase progression and cyclin B1 expression in the NIH3T3 cells (Additional file 3: Figures S3B-S3D), suggesting that induction of G2/M arrest by BTG2 overexpression might be limited to cancer cells which usually contain a defect in G1/S checkpoint, BTG2-mediated MnSOD induction was preceded to p21^WAF1^ expression (Figure 6D) and the expression of p21^WAF1^ was evident both in the RNA and protein levels (Figure 6E and Additional file 4: Figure S4) after transduction and transfection of BTG2 gene in HeLa cells. Furthermore, BTG2 expresser scavenged intracellular H_2_O_2_ level along with cell cycle progression from G1/S to G2/M phase (Figure 6F). All the results can be supported by the notion that NFκB enhances G2/M arrest via induction of p21^WAF1^[38].

## Discussion

**Figure 7:**
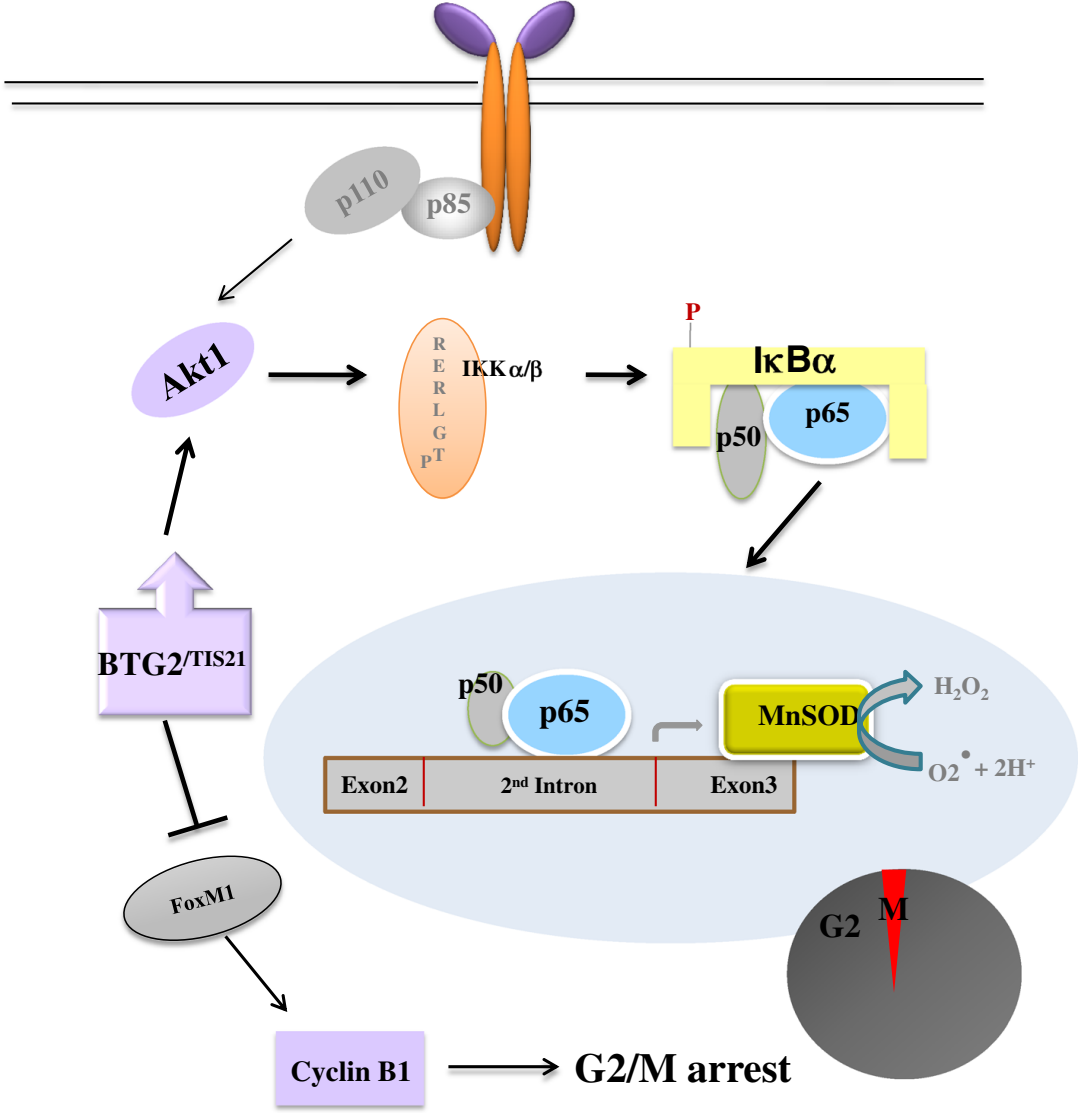
**Crosstalk between PI3K-Akt and NFκB pathways after BTG2 expression in normal and cancer cells via activation of Akt1 and degradation of IκBα protein.** The endogenous and exogenous expressions of BTG2^/TIS21^ significantly induces IκBα degradation via Atk1 activation in cancer and normal cells. The activated NFκB (p65) binds to the enhancer element on the 2^nd^ intron, not promoter, of MnSOD gene and upregulates its transcription in response to BTG2^/TIS21^ expression. The induction of MnSOD expression triggers scavenge of ROS level and the BTG2-mediated ROS reduction inhibits cell cycle progression at G2 phase entry via inhibition of cyclin B1 biosynthesis in contrast to the upregulation of p21^WAF1^ expression.

In the present study, we presented evidences on the crosstalk between PI3K-Akt and NFκB pathways after ectopic and endogenous BTG2 expressions in normal and cancer cells via activation of Akt1 and degradation of IκBα protein. The signals regulating MnSOD expression - the binding of NFκB (p65) to the enhancer element of MnSOD gene in response to BTG2 expression - significantly reduced ROS level and cyclin B1 biosynthesis as opposed to p21^WAF1^ induction, which resulted in G2/M arrest (Figure 7). It has been known that the homeotic expression of MnSOD is regulated by AP-2 and SP-1 binding to the promoter of MnSOD gene [29], whereas induced expression of MnSOD requires enhancer activation located in the 2^nd^ intron [30]. Indeed, the upregulation of BTG2-enhanced MnSOD expression might be one of the intracellular cooperation between APRO gene and a tumor suppressor through the regulations of cell division cycle and intracellular ROS level, since the notion was supported by luciferase assays using promoter and enhancer element of MnSOD gene and by reverse correlations of IκBα and MnSOD expression, in addition to ChIP and immunoblot analyses (Figure 2). Specificity of the effects was further confirmed by RNA interference and IκBα (Ser32, Ser36 to Ala32, Ala36) mutant analysis in HeLa cells (Figure 3) and A549 cells (Figure 5) in addition to the wt-MEF and MCF 7 breast cancer cells (Additional file 2: Figure S2). Our present results are well supported by a recent study that BTG2 works as a coactivator of the antioxidant transcription factor, NFE2L2, which induces antioxidant gene expression including catalase and superoxide dismutases 1 and 2, thus BTG2 is able to protect human mammary epithelial cells from oxidative stress by H_2_O_2_ and other oxidants [39]. In contrast to our present result, HeLa cells have been shown to enhance cell death by H_2_O_2_ burst along with MnSOD induction when the cells are exposed to toxic dose (1.0 μg/ml) of doxorubicin [3]. Therefore, it is highly likely that the BTG2-mediated induction of MnSOD destines cell fate to either G2 arrest or cell death depending on the cellular context. Accordingly, the present study highlights the role of BTG2 as a regulator of ROS level by MnSOD induced via NFκB activation.

BTG2-mediated activation of NFκB via PI3K-Akt pathway was independent of p53 status in normal and cancer cells (Figures 4 and 5, and Additional file 2: Figure S2) such as HeLa (p53 low expresser due to HPV E6 & E7 gene expression), A549, wt-MEF and MCF7 cells (wt-p53), therefore, BTG2-regulated crosstalk between PI3K-Akt1 and NFκB pathways was active in a p53-independent manner. Recently, there are several published reports on the crosstalk; in the growth and survival of primary effusion lymphoma cells [40], malignant invasion and metastasis of NSCLC cells [41], and antiproliferative and proapoptotic effects of metformin in various tumor cells [42]. In addition, DHEA-induced proliferation of ventral prostate epithelial cells and T-cell activation are also finely tuned by NFκB activation via PI3K-Akt [43,44]. However, the above reports did not identify any specific transducer between PI3K-Akt1 and NFκB pathways, whereas we presented here BTG2 as a signal mediator between the two pathways via accelerating IκBα degradation. Nevertheless, how BTG2 induces Akt1 activity still needs to be characterized.

The endogenous expression and activity of MnSOD are significantly downregulated in various cancer cells and tumor tissues as compared with counterpart normal cells [45]. Many studies showed that deficiency of the enzyme activity might be due to the reduction of its transcription. In the present study, we presented for the first time precise signal pathways of MnSOD upregulation by BTG2, in addition to transcriptional regulation of MnSOD expression by enhancer activation of the gene in a BTG2-concentration dependant manner (Figure 1), clearly confirming the involvement of BTG2 in the regulation of MnSOD activity *in vivo*. In the synchronized HeLa cells, BTG2 downregulated cyclin B1 biosynthesis along with G2/M arrest (Figure 6A-6C). The phenomenon is well accordant with our previous report on the disruption of cyclin B1-FoxM1 regulation loop by BTG2 [12]. In addition, sequential inductions of MnSOD and p21^WAF1^ expressions (Figure 6D and 6E) can also be supported by the notion that NFκB enhances G2/M arrest via induction of p21^WAF1^[38]. Even though p21^WAF1^ is a p53 downstream gene, it is also regulated in the p53-independent manner by NFκB activation when the cells are treated with doxorubicin [46], moreover, upstream promoter of p21^WAF1^ contains NFκB response elements [47], supporting that NFκB has been considered as a transcription factor for p21^WAF1^ independent of p53. Fortuitously, all the available data indicate the BTG2-mediated NFκB activation via its interaction with κB-RE and subsequent upregulation of p21^WAF1^ in the p53 nonfunctioning cancer cells.

Depending on the level of ROS, cellular responses are quite variable, ranging from transient to permanent growth arrest, or the cell cycle progression. The series of changes observed in the synchronized HeLa cells (Figure 6), *e.g.* delayed biosynthesis of cyclin B1 along with delayed entry into G2 phase at 4 h, increase of MnSOD expression at 6 h, significant reduction of H_2_O_2_ level at 7 h, and then p21^WAF1^ induction and G2/M phase arrest at 8 h after the release from thymidine double blocks reflect a mechanism of APRO activity exhibited by BTG2. Nonetheless, cellular response to BTG2 expression would be diverse, depending on the cellular context and its ROS level.

## Conclusions

It is well documented that cancer cells contain defective mitochondria and often reprogram their metabolic pathways to meet with their energy requirements during the process of tumor progression [48]. The major metabolic phenotype described in Warburg effect is the shift of ATP generation from oxidative phosphorylation to glycolysis under physiologic oxygen concentration [49]. Here, MnSOD plays a critical role in the maintenance of mitochondrial integrity, because cells lacking MnSOD are defective in ATP generation by impairing oxidative phosphorylation. Therefore, MnSOD has been regarded as a guardian of powerhouse in tumorigenesis models [50]. Low level of MnSOD may create threat to mitochondrial function at the early stages of carcinogenesis [51], therefore, cancer cells contain defective mitochondria due to lower expression of MnSOD. In that sense, it is worth to note that BTG2-induced MnSOD expression might be one of the early events of tumor suppression by maintaining the integrity of mitochondria. We have recently observed that exogenously expressed BTG2 was rapidly translocated to mitochondria in response to H_2_O_2_ in H9c2 cardiomyoblasts [52]. Therefore, we speculate that BTG2 might play a significant role in the modulation of mitochondrial defect in cancer cells. *In vivo* studies using BTG2 knockout mice might be helpful in finding the significance of BTG2 in carcinogenesis.

## Materials and methods

### Cell treatment reagents and plasmids

HeLa cells were cultured in DMEM medium supplemented with 10% heat inactivated fetal bovine serum (FBS) in a humidified atmosphere containing 5% CO_2_ at 37°C. Wild type mouse embryo fibroblast (MEF) were prepared in our laboratory with 13.5 day old embryos isolated from wild type and BTG2^/TIS21-/-^ mice, and then cultured in DMEM with 10% FBS. Antibodies against α-tubulin, hemagglutinin (HA), IκBα, pIKKα/β-Ser176, pAkt, cyclinB1, p21^WAF1^ and BTG2 were purchased from Santa Cruz (Santa Cruz, CA), glutathione peroxidase (GPX), SOD1, MnSOD and catalase were from the Lab Frontier (*Lab Frontier* Life Science Institute, Seoul, Korea), Akt2 was from Upstate Biotechnology (Lake Placid, NY), and Akt, pFoxO3a-Ser253 and BCL-xL were purchased from Cell Signaling Technology Inc. (Danvers, MA). Anti β-actin antibody, LY294002, cycloheximide, Wortmanin and MG-132 were purchased from Sigma (St. Louis, MO). Cell treatment times and concentrations were mentioned in legends for figures. The BTG2^/TIS21^ were inserted into the *Eco*RI and *Xho*I sites of the pcDNA_3_-HA vector. DNA sequences of BTG2^/TIS21^ in pcDNA_3_-HA were verified by automatic sequence analysis (ABI 377). Full-length SP1 were cloned into the EcoRI and SalI sites of p3xFLAG-CMV (Sigma).

### Adenoviral transduction of HeLa cells with BTG2 gene

*Ad*-*BTG2* virus was prepared in our laboratory according to the method described previously [3,12,32] and infected to HeLa cells for 5 h, and then incubated in the complete media for 48 h until subjected to various analyses. Infection of Ad*-LacZ* was employed as the control of adenoviral transduction.

### Cloning of κB-response element (κB-RE) into pGL3 basic vector

Cloning of enhancer element of MnSOD gene was performed in our laboratory by PCR amplification using human genomic DNAs isolated from HeLa cells as the template, and the upstream and downstream primers were obtained from the 2^nd^ intron of MnSOD gene, 5′ ACCTCGAGTGATTGTGTTTGAAGTAAATG-3′ and 5′ AAAAAGCTTTGATTCCACAAGTAAAGG-3′, respectively. PCR amplification was performed using *pfu* polymerase (Takara Inc., Japan) according to the protocol; Denaturation at 95°C for 30s, annealing at 55°C for 30s, and elongation at 72°C for 60s. The amplified PCR products were digested with XhoI and HindIII, and then subcloned into pGL3 basic luciferase reporter vector purchased from Promega (Madison, WI) using the same sites. The recombinant DNA sequences, κB-RE, were confirmed by nucleotide sequencing (Genotech Corp., Daejeon, Korea).

### Transfection analyses

Increasing amounts of BTG2^/TIS21^ cDNAs were co-transfected with either promoter construct or κB-RE of MnSOD gene using Metafectane reagent (BionTex, Munich, Germany) and thymidine kinase promoter-driven Renilla luciferase plasmid as a control DNA. The activity of luciferase was measured by TD 20/20 luminometer (Turner BioSystems, Sunnyvale, CA) according to the instructions for the Dual-Luciferase Reporter Assay System (Promega, Madison, WI). All transfection experiments and luciferase assays were carried out in triplicate and repeated more than twice. Reporter construct containing MnSOD promoter (−2215 to +42) was a gift from Dr. Curtis Harris in NIH, USA.

### Chromatin immunoprecipitation (ChIP) assay

Binding of activated NFκB to the κB-RE was analyzed by ChIP assay. Cells fixed with 1% formaldehyde were washed twice using ice-cold PBS containing protease inhibitors (1.0 mM PMSF, 1.0 μg/ml aprotinin, and 1.0 μg/ml pepstatin), and the cell pellets collected by centrifugation at 5,000 × g for 5 min at 4°C were resuspended in the lysis buffer (50 mM Tris–HCl, pH 8.1, 10 mM EDTA, and 1.0% SDS) containing protease inhibitors. Cross-linked chromatin was sonicated on ice to shear DNA to 200 to 1000 bp length and then centrifuged at 13,000 × g for 10 min to remove cell debris. The soluble chromatin was then diluted 10-fold in ChIP dilution buffer (16.7 mM Tris, 167 mM NaCl, 1.1% Triton X-100, and 0.01% SDS). The diluted soluble chromatin fraction was pre-cleaned by 30 μl protein G-agarose beads. Pre-cleaned chromatin was mixed with the anti-p65 antibody (ChIP grade, Santa Cruz) overnight at 4°C. Parallel control experiments were also performed with pre-immune control precipitates using unstimulated IgG. The antibody-chromatin complex was pulled-down with protein G beads for 2 h rotation at 4°C. Protein G beads were then washed by rotation at 4°C with 400 μl of buffer in the following order; low salt immune complex wash buffer (20 mM Tris–HCl, pH 8.1, 150 mM NaCl, 2 mM EDTA, 1% Triton X-100, 1% SDS), high salt immune complex wash buffer (20 mM Tris–HCl, pH 8.1, 500 mM NaCl, 2 mM EDTA, 1.0% Triton X-100, 1.0% SDS), LiCl immune complex wash buffer (10 mM Tris–HCl, pH 8.1, 0.25 mM LiCl, 1.0% deoxycholate, 1.0% Nonidet P-40, 0.1% SDS), followed by two washes with 10 mM Tris–HCl, pH 8.1 with 1.0 mM EDTA, pH 8.1. Precipitated immune complexes were eluted twice with 250 μl of elution buffer (1.0% SDS, 0.1 M NaHCO_3_) at room temperature for 15 min each. Reversal of cross-linking was performed by adding 20 μl of 5 M NaCl to the elution mixture and heating at 65°C overnight. The elutes were mixed with 0.5 M EDTA, 1.0 M Tris (pH 6.5) and 2 μl of 10 mg Proteinase K and shaken well. DNAs recovered by phenol:chloroform extraction and ethanol precipitation were resuspended in nuclease-free water for PCR amplification using the primers described in the Additional file 5.

### Measurement of the level of reactive oxygen species

The intracellular H_2_O_2_ concentration was measured by FACS analysis using 20 μM 2′,7′-dichlorodihydrofluorescein diacetate (H_2_-DCFDA, Molecular Probes). Cells infected with Ad-BTG2 or Ad-LacZ were pretreated with H_2_-DCFDA for 10 min and then the fluorescence of dichlorodihydrofluorescein derived from oxidation of H_2_-DCFDA was measured.

### Small interfering RNAs (siRNAs) and preparation of lentivirus with IκBα mutant

The five sets of siBTG2 sequences and one set of siAtk1 used in the experiments are described in the Additional file 6. The mixture containing siRNAs was prepared with a concentration of 4 μM each and used for transfection of normal and cancer cells using Lipofectamine 2000 (Invitrogen, Carlsbad, CA) according to the manufacturer’s instruction. Lentivirus with IκBα-mutant was prepared in 293 T cells by cotransfection of IκBα-mutant (Ser32, Ser36 to Ala32, Ala36) and pCMV-VSV-G helper constructs using Lipofectamine 2000. Viral supernatants were harvested in 48 h and filtered through a 0.22 μm filter (Millipore). HeLa cells (2 × 10^5^/60 mm-diameter) were transduced with 250 μl of the supernatant. Culture medium was changed in 12 h and maintained for 2 days until analysis.

### RT-PCR

Total cellular RNAs (1.0 μg) isolated with RNAiso Plus were used for cDNA preparation and then amplified by PCR kit (Takara Inc., Japan); First strand cDNA was synthesized using oligo-dT by reverse transcription reaction in 10 μl of reaction volume. The gene of interest was amplified by ExTaq polymerase in PCR kits using primer sequences described in the Additional file 5.

### Immunoblot analyses

Cells were sonicated with RIPA buffer [50 mM Tris/HCl, pH 7.5, 150 mM NaCl, 1.0% Nonidet P-40, 0.1% SDS, 0.5% deoxycholic acid, 50 mM sodium fluoride, 1.0 mM sodium vanadate, 1.0 mM phenylmethylsulfonyl fluoride (PMSF), 1.0 μg/ml leupeptin], and 40 μg of cell lysates were resolved on 8 to 12% SDS-PAGE in 25 mM Tris-glycine buffer. The gel-resolved proteins were then transferred to nitrocellulose membrane. The membranes were blocked with 5% nonfat skim milk in PBS containing 0.05% Tween 20 (PBST) for 1 h and then incubated with respective antibodies overnight at 4°C. Nitrocellulose membranes were washed three times with PBST and then incubated with horseradish peroxidase-conjugated secondary antibodies for 1 h. ECL (Amersham Biosciences, UK) kit was employed to visualize protein expression levels. Protein bands were quantified by relative optical densities using Image J software.

### Cell synchronization and FACS analysis

HeLa cells were synchronized with 1.0 mM thymidine treatment for 20 h, released for 9 h and then treated again with thymidine for 13 h more before release from the treatment. In the meantime, cells were infected with 100 moi of Ad-TIS21 or Ad-LacZ for 5 h. The experimental time points are further explained in detail in the Additional file 3: Figure S3A. Cell cycle analysis was performed by flow cytometry (BD FACScan II, BD Biosciences, San Jose, CA) after staining the DNA content with propidium iodide (Sigma) according to the manufacturer’s instruction. Cell cycle phases were analyzed by ModFit software.

## Abbreviations

BTG2: B-cell translocation gene 2; TIS21: TPA-inducible sequences 21; MnSOD: Manganese superoxide dismutase; IκB: Inhibtior of NF-κB; NF-κB: Nuclear factor kappa B; IKK: IκB Kinase; ROS: Reactive oxygen species; MEF: Mouse embryonic fibroblast.

## Competing interests

The authors declared that they have no competing interests.

## Authors’ contributions

SS performed the experiments and MSR cloned the kB-RE for Luciferase analysis, prepared all the necessary stuffs and contributed for valuable discussion. IKL conceived the study and guided the design of experiments. SS drafted the manuscript and IKL edited it and writing the article. All authors read and approved the final manuscript.

## Supplementary Material

Additional file 1: Figure S1 **(A)** HeLa cells (2 × 10^5^) were seeded in 60 mm dish and maintained for 12 h. Transfection of the cells with BTG2-HA (0.8 μg of DNA) or control vector (0.8 μg of DNA) was performed for 6 h, and followed by media change. In 48 h, cells were harvested for immunoblot analysis using anti-pFOXO3a antibody. α -tubulin was used as a loading control. **(B)** HeLa cells were treated with TPA 100 ng for 2 h and analyzed for IκBα degradation. Click here for file

Additional file 2: Figure S2 **(A)** MCF7 cells (2 × 10^5^) were seeded in 60 mm dish and maintained for 12 h, and then subjected to transfection for 6 h with BTG2 cDNA (0 ~ 0.8 μg) until media change. Equal DNA content was adjusted with the control vector. In 48 h, cells were harvested for immunoblot analysis and examined the degradation of IκBα by transfection of BTG2. **(B)** ChIP assay; the above treatment revealed specific interaction of p65 to kB-RE only in the BTG2 expressers. **(C)** To confirm the effect of BTG2 expression on IκBα degradation not only in cancer cells but also in normal cells, wild type mouse embryo fibroblasts (MEF) were transfected with siBTG2 (~100 nM), and then accumulation of IκBα was examined by immunoblot analysis along with knockdown of BTG2 expression by RT-PCR. Click here for file

Additional file 3: Figure S3 **(A)** Schema of cell synchronization at G1/S boundary. NIH3T3 cells (2 × 10^5^) were seeded in 60 mm dish and infected with either Ad-BTG2 virus (100 moi) or Ad-LacZ for 5 h. In 9 h, the cells were treated with 2.5 mM thymidine for 12 h and then released for 12 h by media change. Finally, the cells were harvested at the various time points for FACS analysis to examine DNA content by staining with propidium iodide. **(B)** NIH3T3 (2 × 10^5^) cells synchronized by thymidine treatment twice were harvested at 0, 4, 8 and 12 h and then subjected to PI staining for FACS anlalysis. Note absence of any difference in the G2/M phase progression between the Ad-BTG2 (100 moi) or Ad-LacZ infected groups. **(C)** Quantification of each cell cycle phases observed in the NIH3T3 cells infected with either Ad-BTG2 or Ad-LacZ virus along with thymidine double block. No significant difference in the progression of G2/M phase progression between the two groups. **(D)** Immunoblot analysis showing the similar progression of G2/M phase, monitored by cyclin B1 synthesis and degradation. Click here for file

Additional file 4: Figure S4 HeLa cells (2 × 10^5^) were seeded in 60 mm dish and maintained for 12 h. Cells were transfected with BTG2 cDNA (0.8 μg) and control vector (0.8 μg) for 6 h, followed by media change. In 48 h, cells were harvested for immunoblot analysis to check for upregulation of p21^WAF1^ protein induced by BTG2. α-tubulin was used as a loading control. Click here for file

Additional file 5 Primer sequences for RT-PCR, ChIP assay, and gene cloning analyses in human cells. Click here for file

Additional file 6 RNA sequences used for interference of BTG2 expression in human cells. Click here for file
