# Supplementary material for: B-cell translocation gene 2 mediates crosstalk between PI3K/Akt1 and NFκB pathways which enhances transcription of MnSOD by accelerating IκBα degradation in normal and cancer cells
Source: Cell Commun Signal. 2013 Sep 18;11:69. doi: 10.1186/1478-811X-11-69 (PMC3851984; doi:10.1186/1478-811X-11-69)
Supplement: Additional file 1: Figure S1 — (A) HeLa cells (2 × 10^5^) were seeded in 60 mm dish and maintained for 12 h. Transfection of the cells with BTG2-HA (0.8 μg of DNA) or control vector (0.8 μg of DNA) was performed for 6 h, and followed by media change. In 48 h, cells were harvested for immunoblot analysis using anti-pFOXO3a antibody. α -tubulin was used as a loading control. (B) HeLa cells were treated with TPA 100 ng for 2 h and analyzed for IκBα degradation. [file 1478-811X-11-69-S1.pptx]

## Slide 1
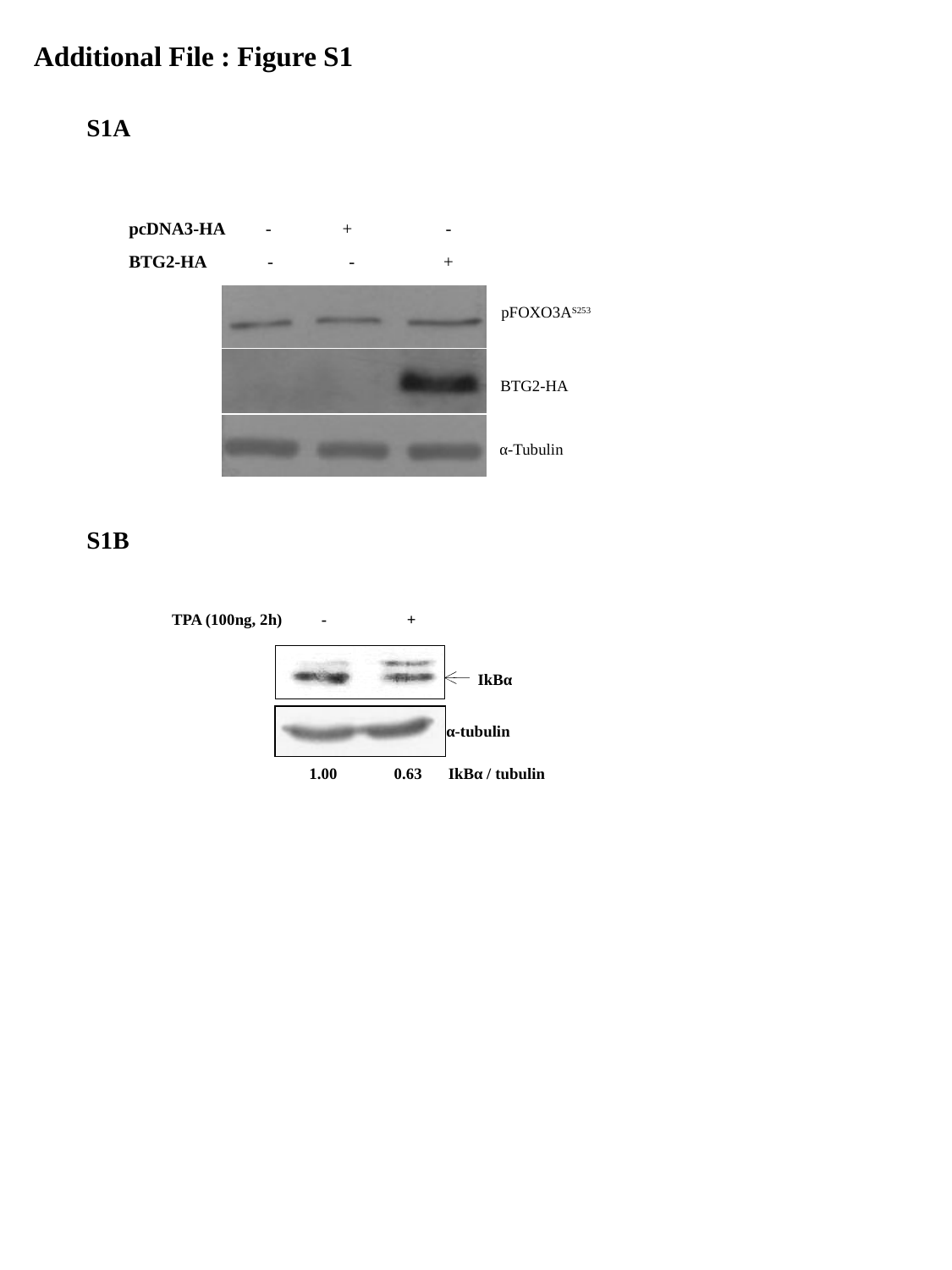

Additional File : Figure S1
S1A
pcDNA3-HA
 - + -
BTG2-HA
 - - +
pFOXO3AS253
BTG2-HA
α-Tubulin
S1B
TPA (100ng, 2h)
 - +
IkBα
α-tubulin
1.00
0.63
IkBα / tubulin
