# Supplementary material for: B-cell translocation gene 2 mediates crosstalk between PI3K/Akt1 and NFκB pathways which enhances transcription of MnSOD by accelerating IκBα degradation in normal and cancer cells
Source: Cell Commun Signal. 2013 Sep 18;11:69. doi: 10.1186/1478-811X-11-69 (PMC3851984; doi:10.1186/1478-811X-11-69)
Supplement: Additional file 2: Figure S2 — (A) MCF7 cells (2 × 10^5^) were seeded in 60 mm dish and maintained for 12 h, and then subjected to transfection for 6 h with BTG2 cDNA (0 ~ 0.8 μg) until media change. Equal DNA content was adjusted with the control vector. In 48 h, cells were harvested for immunoblot analysis and examined the degradation of IκBα by transfection of BTG2. (B) ChIP assay; the above treatment revealed specific interaction of p65 to kB-RE only in the BTG2 expressers. (C) To confirm the effect of BTG2 expression on IκBα degradation not only in cancer cells but also in normal cells, wild type mouse embryo fibroblasts (MEF) were transfected with siBTG2 (~100 nM), and then accumulation of IκBα was examined by immunoblot analysis along with knockdown of BTG2 expression by RT-PCR. [file 1478-811X-11-69-S2.pptx]

## Slide 1
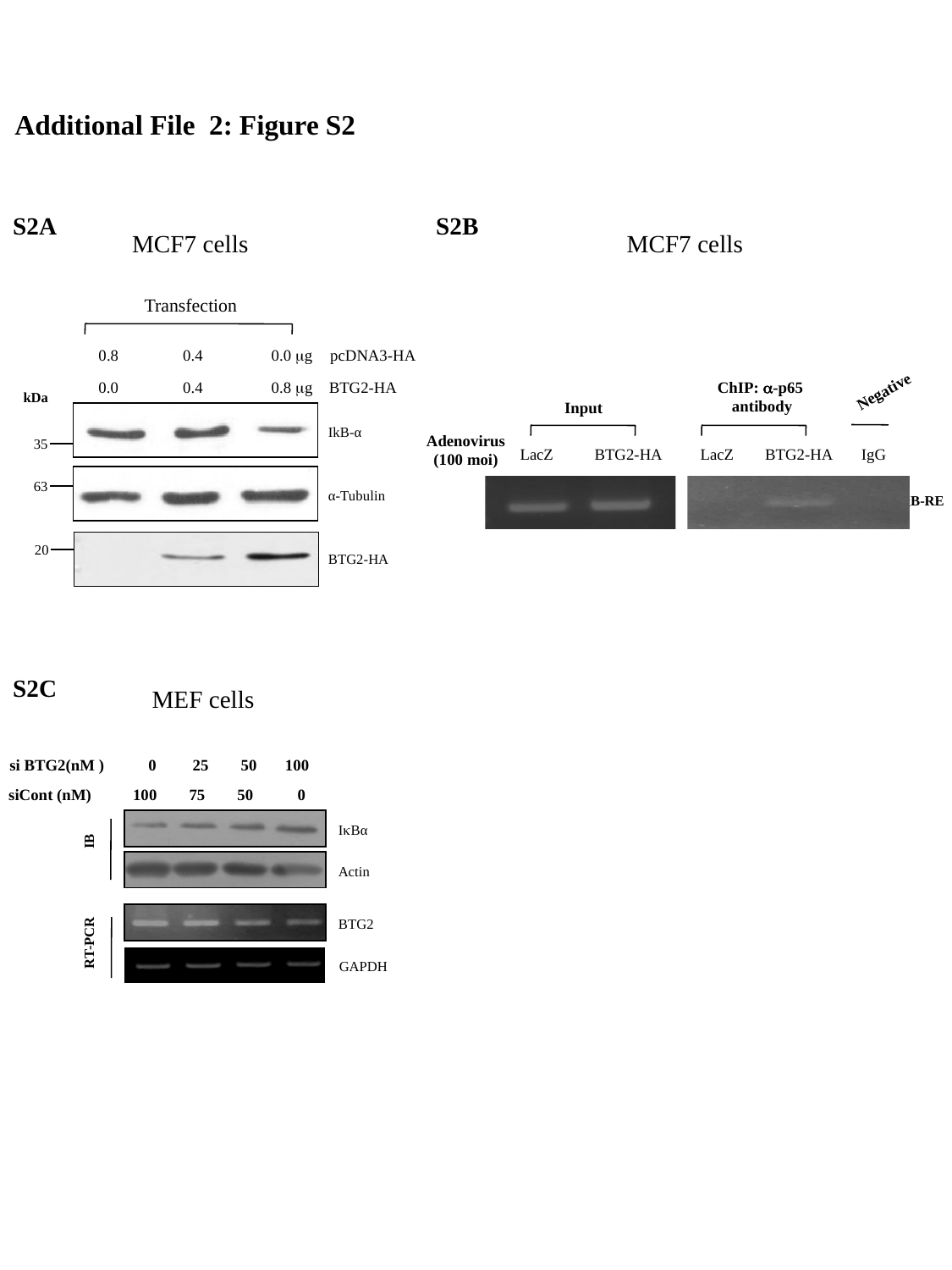

Additional File 2: Figure S2
S2A
S2B
MCF7 cells
MCF7 cells
Transfection
 0.8 0.4 0.0 mg
pcDNA3-HA
 0.0 0.4 0.8 mg
BTG2-HA
kDa
IkB-α
35
63
α-Tubulin
20
BTG2-HA
Negative
ChIP: a-p65
antibody
Input
Adenovirus
(100 moi)
LacZ
BTG2-HA
LacZ
BTG2-HA
IgG
 kB-RE
S2C
MEF cells
si BTG2(nM )
 0 25 50 100
siCont (nM)
100 75 50 0
IkBα
IB
Actin
RT-PCR
BTG2
GAPDH
