# Supplementary material for: B-cell translocation gene 2 mediates crosstalk between PI3K/Akt1 and NFκB pathways which enhances transcription of MnSOD by accelerating IκBα degradation in normal and cancer cells
Source: Cell Commun Signal. 2013 Sep 18;11:69. doi: 10.1186/1478-811X-11-69 (PMC3851984; doi:10.1186/1478-811X-11-69)
Supplement: Additional file 3: Figure S3 — (A) Schema of cell synchronization at G1/S boundary. NIH3T3 cells (2 × 10^5^) were seeded in 60 mm dish and infected with either Ad-BTG2 virus (100 moi) or Ad-LacZ for 5 h. In 9 h, the cells were treated with 2.5 mM thymidine for 12 h and then released for 12 h by media change. Finally, the cells were harvested at the various time points for FACS analysis to examine DNA content by staining with propidium iodide. (B) NIH3T3 (2 × 10^5^) cells synchronized by thymidine treatment twice were harvested at 0, 4, 8 and 12 h and then subjected to PI staining for FACS anlalysis. Note absence of any difference in the G2/M phase progression between the Ad-BTG2 (100 moi) or Ad-LacZ infected groups. (C) Quantification of each cell cycle phases observed in the NIH3T3 cells infected with either Ad-BTG2 or Ad-LacZ virus along with thymidine double block. No significant difference in the progression of G2/M phase progression between the two groups. (D) Immunoblot analysis showing the similar progression of G2/M phase, monitored by cyclin B1 synthesis and degradation. [file 1478-811X-11-69-S3.pptx]

## Slide 1
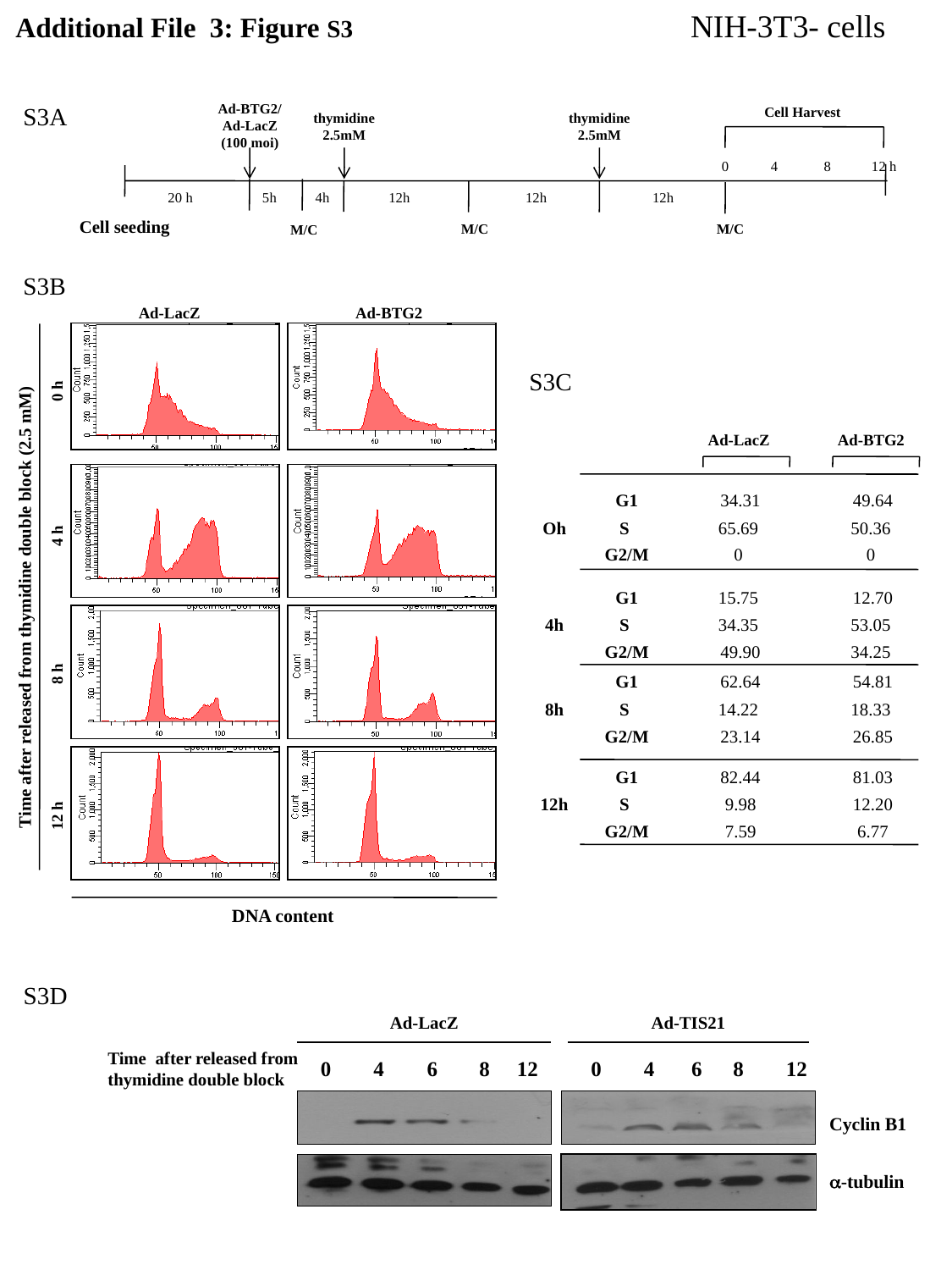

NIH-3T3- cells
Additional File 3: Figure S3
Ad-BTG2/
Ad-LacZ
(100 moi)
Cell Harvest
thymidine
2.5mM
thymidine
2.5mM
0
4
8
12 h
20 h
5h
4h
12h
12h
12h
Cell seeding
M/C
M/C
S3A
M/C
S3B
Ad-LacZ
Ad-BTG2
0 h
4 h
Time after released from thymidine double block (2.5 mM)
8 h
12 h
DNA content
S3C
Ad-LacZ
Ad-BTG2
G1
34.31
49.64
Oh
S
65.69
50.36
G2/M
0
0
G1
15.75
12.70
S
34.35
53.05
4h
G2/M
49.90
34.25
G1
62.64
54.81
S
14.22
18.33
8h
G2/M
23.14
26.85
G1
82.44
81.03
12h
S
9.98
12.20
G2/M
7.59
6.77
S3D
Ad-LacZ
Ad-TIS21
Time after released from thymidine double block
0
4
6
8
12
0
4
6
8
12
Cyclin B1
α-tubulin
