# Supplementary figures and images for: B-cell translocation gene 2 mediates crosstalk between PI3K/Akt1 and NFκB pathways which enhances transcription of MnSOD by accelerating IκBα degradation in normal and cancer cells
Source: Cell Commun Signal. 2013 Sep 18;11:69. doi: 10.1186/1478-811X-11-69 (PMC3851984; doi:10.1186/1478-811X-11-69)

## Slide 1
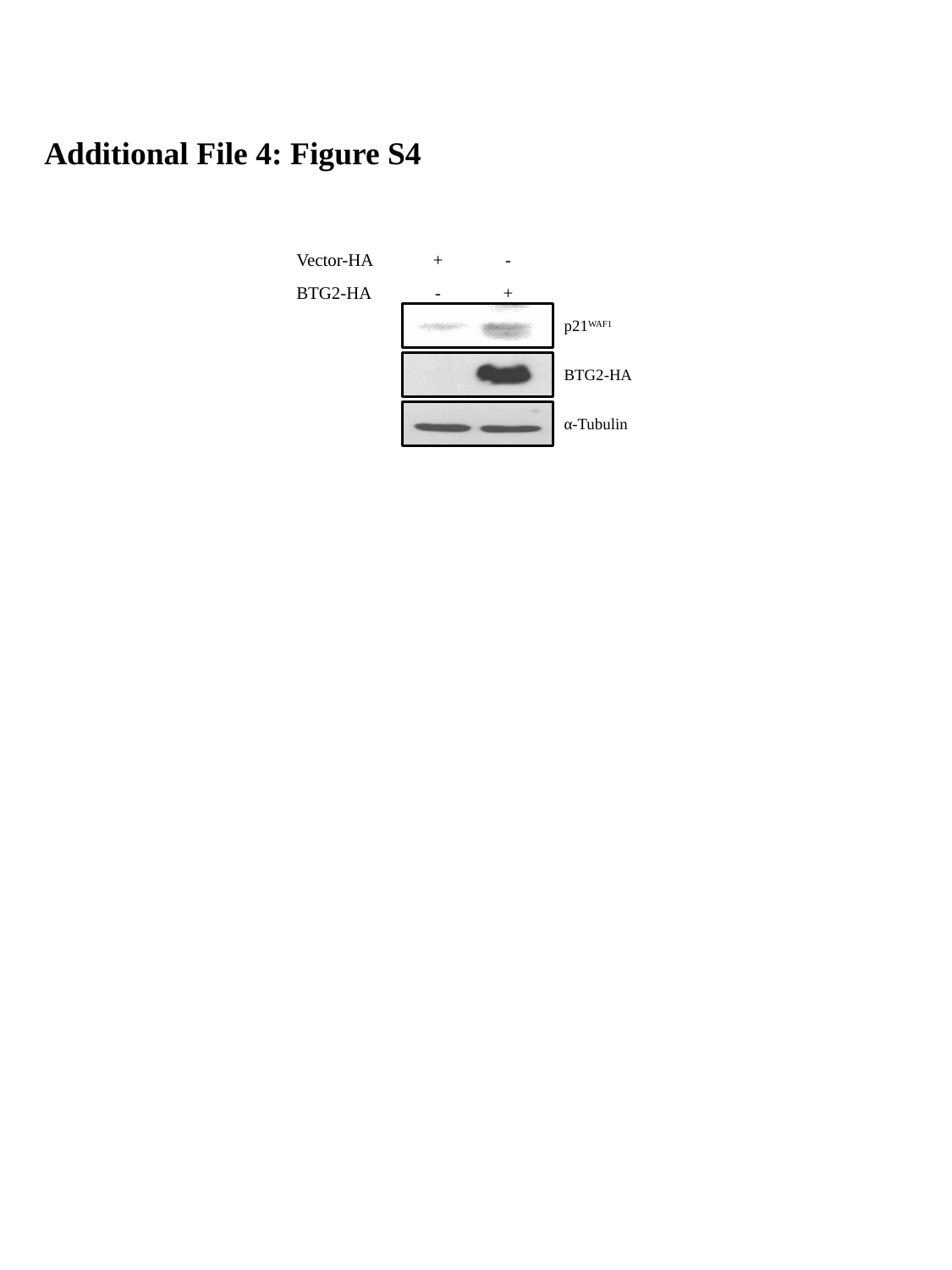

Additional File 4: Figure S4
Vector-HA
 + -
BTG2-HA
 - +
p21WAF1
BTG2-HA
α-Tubulin

Supplement: Additional file 4: Figure S4 — HeLa cells (2 × 10^5^) were seeded in 60 mm dish and maintained for 12 h. Cells were transfected with BTG2 cDNA (0.8 μg) and control vector (0.8 μg) for 6 h, followed by media change. In 48 h, cells were harvested for immunoblot analysis to check for upregulation of p21WAF1 protein induced by BTG2. α-tubulin was used as a loading control. [file 1478-811X-11-69-S4.pptx]
