# Supplementary material for: B-cell translocation gene 2 mediates crosstalk between PI3K/Akt1 and NFκB pathways which enhances transcription of MnSOD by accelerating IκBα degradation in normal and cancer cells
Source: Cell Commun Signal. 2013 Sep 18;11:69. doi: 10.1186/1478-811X-11-69 (PMC3851984; doi:10.1186/1478-811X-11-69)
Supplement: Additional file 5 — Primer sequences for RT-PCR, ChIP assay, and gene cloning analyses in human cells. [file 1478-811X-11-69-S5.pptx]

## Slide 1
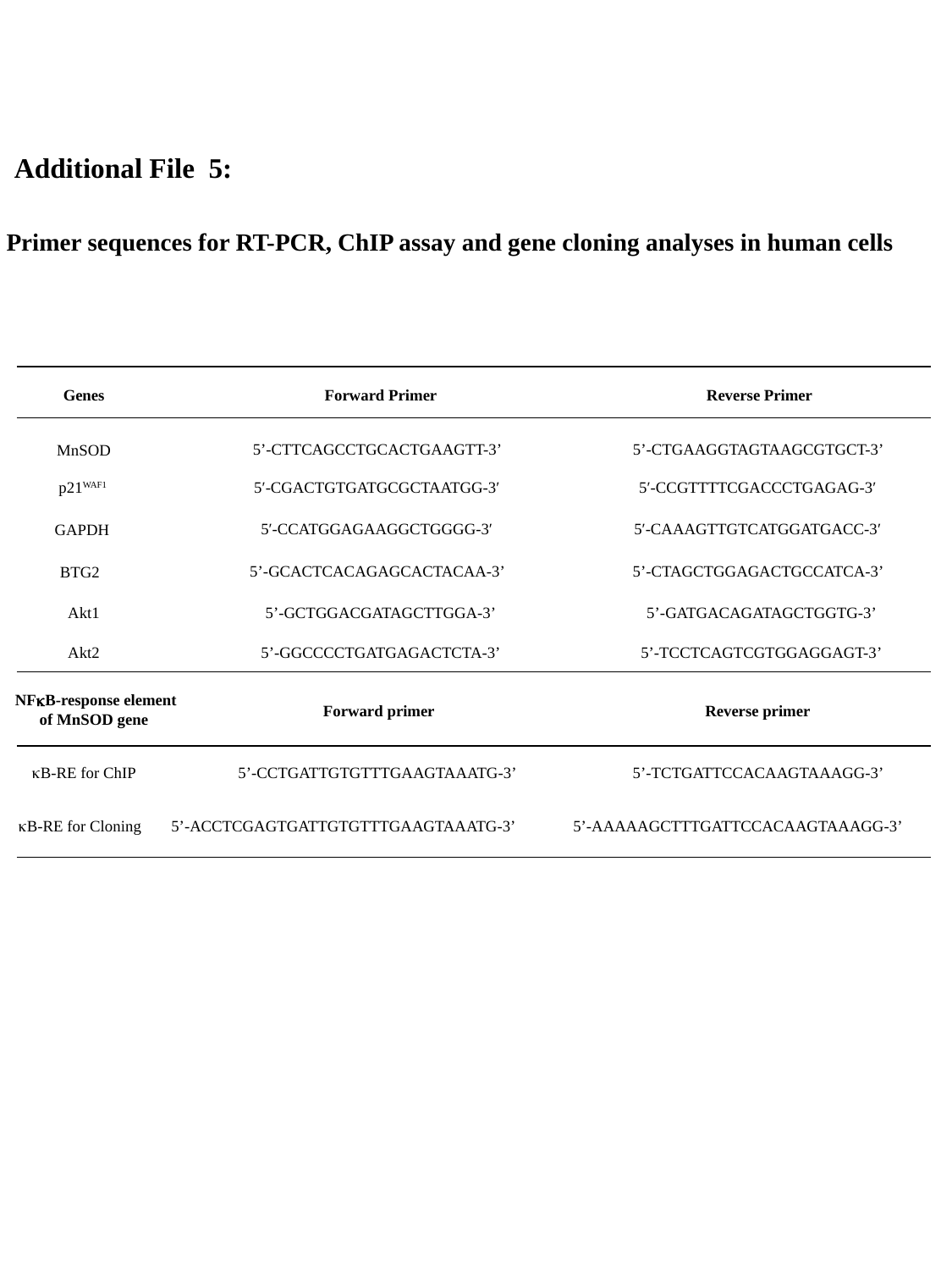

Additional File 5:
Primer sequences for RT-PCR, ChIP assay and gene cloning analyses in human cells
Genes
Forward Primer
Reverse Primer
MnSOD
5’-CTTCAGCCTGCACTGAAGTT-3’
5’-CTGAAGGTAGTAAGCGTGCT-3’
p21WAF1
5′-CGACTGTGATGCGCTAATGG-3′
5′-CCGTTTTCGACCCTGAGAG-3′
GAPDH
5′-CCATGGAGAAGGCTGGGG-3′
5′-CAAAGTTGTCATGGATGACC-3′
BTG2
5’-GCACTCACAGAGCACTACAA-3’
5’-CTAGCTGGAGACTGCCATCA-3’
Akt1
5’-GCTGGACGATAGCTTGGA-3’
5’-GATGACAGATAGCTGGTG-3’
Akt2
5’-GGCCCCTGATGAGACTCTA-3’
5’-TCCTCAGTCGTGGAGGAGT-3’
NFkB-response element
of MnSOD gene
Forward primer
Reverse primer
kB-RE for ChIP
5’-CCTGATTGTGTTTGAAGTAAATG-3’
5’-TCTGATTCCACAAGTAAAGG-3’
kB-RE for Cloning
5’-ACCTCGAGTGATTGTGTTTGAAGTAAATG-3’
5’-AAAAAGCTTTGATTCCACAAGTAAAGG-3’
