# Supplementary material for: B-cell translocation gene 2 mediates crosstalk between PI3K/Akt1 and NFκB pathways which enhances transcription of MnSOD by accelerating IκBα degradation in normal and cancer cells
Source: Cell Commun Signal. 2013 Sep 18;11:69. doi: 10.1186/1478-811X-11-69 (PMC3851984; doi:10.1186/1478-811X-11-69)
Supplement: Additional file 6 — RNA sequences used for interference of BTG2 expression in human cells. [file 1478-811X-11-69-S6.pptx]

## Slide 1
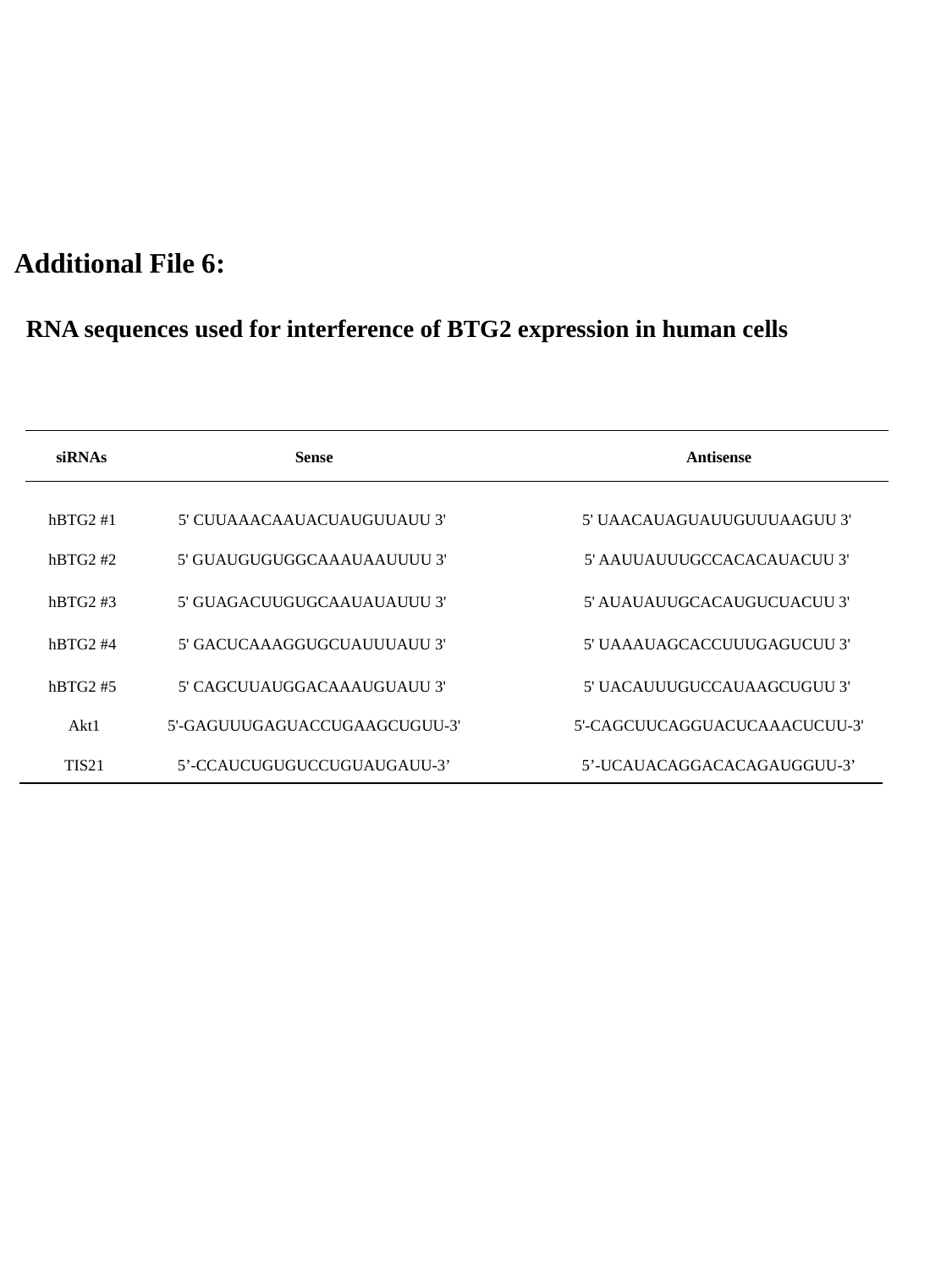

Additional File 6:
RNA sequences used for interference of BTG2 expression in human cells
siRNAs
Sense
Antisense
hBTG2 #1
5' CUUAAACAAUACUAUGUUAUU 3'
5' UAACAUAGUAUUGUUUAAGUU 3'
hBTG2 #2
5' GUAUGUGUGGCAAAUAAUUUU 3'
5' AAUUAUUUGCCACACAUACUU 3'
hBTG2 #3
5' GUAGACUUGUGCAAUAUAUUU 3'
5' AUAUAUUGCACAUGUCUACUU 3'
hBTG2 #4
5' GACUCAAAGGUGCUAUUUAUU 3'
5' UAAAUAGCACCUUUGAGUCUU 3'
hBTG2 #5
5' CAGCUUAUGGACAAAUGUAUU 3'
5' UACAUUUGUCCAUAAGCUGUU 3'
Akt1
5'-GAGUUUGAGUACCUGAAGCUGUU-3'
5'-CAGCUUCAGGUACUCAAACUCUU-3'
TIS21
5’-CCAUCUGUGUCCUGUAUGAUU-3’
5’-UCAUACAGGACACAGAUGGUU-3’
